# Hexakis(1*H*-imidazole-κ*N* ^3^)cobalt(III) tris­(hexa­fluoridophosphate) hexa­hydrate

**DOI:** 10.1107/S160053680903863X

**Published:** 2009-09-30

**Authors:** Andrzej Surdykowski, Anna Łęczkowska, Liliana Dobrzańska, Edward Szłyk

**Affiliations:** a Faculty of Chemistry, Nicolaus Copernicus University, Gagarina 7, 87-100 Toruń, Poland; b Department of Chemistry, University of Stellenbosch, Private Bag X1, Matieland 7602, South Africa

## Abstract

In the crystal structure of the title compound, [Co(C_3_H_4_N_2_)_6_](PF_6_)_3_·6H_2_O, the Co^III^ atom lies on a special position with site-symmetry 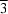 and the P atom is located on a special position with site symmetry 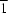. The Co^III^ atom has an almost ideal octa­hedral coordination formed by the N atoms of six imidazole ligands. The water mol­ecules form hydrogen-bonded helical chains propagating in [001] by O—H⋯O inter­actions with a distance of 2.913 (2) Å. They simultaneously inter­act as hydrogen-bond acceptors and donors with the cations and anions, respectively, resulting in the formation of a three-dimensional assembly. Weak C—H⋯F inter­actions further stabilize the crystal structure.

## Related literature

For Co^III^ complexes with heterocycles, see: Wojtczak *et al.* (1990 ▶); Pazderski *et al.* (2008 ▶). For the hexa­kis(imidazole)-cobalt(III) ion in solution, see: Navon & Panigel (1989 ▶); Wiśniewska & Kita (2006 ▶). For Co—N bond distances in hexa­kis(imidazole)-cobalt(II) complexes, see: Tong *et al.* (2002 ▶). For Co^III^—N and Co^II^—N bond lengths in hexa­ammine–cobalt complexes, see: Kime & Ibers (1969 ▶). The water mol­ecules present in the crystal structure form helical chains similar to those observed in a trichloro­phloroglucinol structure, see: Saha & Nangia (2005 ▶).
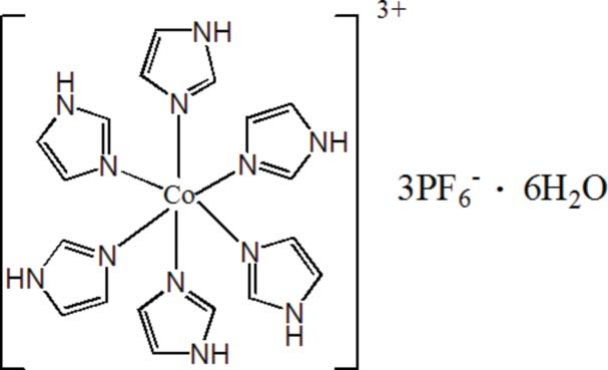


## Experimental

### 

#### Crystal data

[Co(C_3_H_4_N_2_)_6_](PF_6_)_3_·6H_2_O
*M* *_r_* = 1010.43
Trigonal, 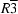

*a* = 20.9911 (13) Å
*c* = 7.0156 (9) Å
*V* = 2677.1 (4) Å^3^
*Z* = 3
Mo *K*α radiation
μ = 0.77 mm^−1^
*T* = 100 K
0.25 × 0.25 × 0.21 mm

#### Data collection

Bruker SMART APEX CCD area-detector diffractometer
Absorption correction: multi-scan (*SADABS*; Sheldrick, 1997 ▶) *T*_min_ = 0.832, *T*_max_ = 0.856
4893 measured reflections
1373 independent reflections
1288 reflections with *I* > 2σ(*I*)
*R*_int_ = 0.026

#### Refinement

*R*[*F* ^2^ > 2σ(*F* ^2^)] = 0.031
*wR*(*F* ^2^) = 0.083
*S* = 1.08
1373 reflections
98 parameters
3 restraints
H atoms treated by a mixture of independent and constrained refinement
Δρ_max_ = 0.54 e Å^−3^
Δρ_min_ = −0.31 e Å^−3^

### 

Data collection: *SMART* (Bruker, 2001 ▶); cell refinement: *SAINT* (Bruker, 2002 ▶); data reduction: *SAINT*; program(s) used to solve structure: *SHELXS97* (Sheldrick, 2008 ▶); program(s) used to refine structure: *SHELXL97* (Sheldrick, 2008 ▶); molecular graphics: *X-SEED* (Barbour 2001 ▶); software used to prepare material for publication: *SHELXL97*.

## Supplementary Material

Crystal structure: contains datablocks I, global. DOI: 10.1107/S160053680903863X/hg2572sup1.cif

Structure factors: contains datablocks I. DOI: 10.1107/S160053680903863X/hg2572Isup2.hkl

Additional supplementary materials:  crystallographic information; 3D view; checkCIF report

## Figures and Tables

**Table 1:** Hydrogen-bond geometry (Å, °)

*D*—H⋯*A*	*D*—H	H⋯*A*	*D*⋯*A*	*D*—H⋯*A*
O1—H6⋯O1^i^	0.96 (3)	1.98 (3)	2.913 (2)	163 (3)
N3—H3⋯O1	0.88	1.98	2.834 (2)	165
O1—H7⋯F3^ii^	0.96 (3)	2.10 (3)	2.945 (2)	146 (2)
C2—H2⋯F3^iii^	0.95	2.34	3.042 (2)	131
C4—H4⋯F1^iv^	0.95	2.40	3.303 (2)	158

Symmetry codes: (i) 
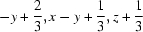
; (ii) 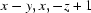; (iii) 
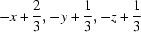
; (iv) 
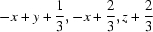
.

**Fig. 1.**
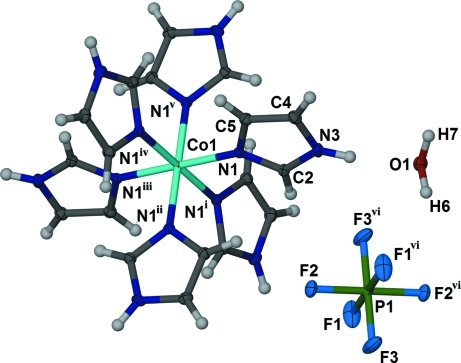
The molecular structure of (I), with the atom-numbering scheme [symmetry codes:(i) y,-x + y,-z; (ii) -x + y, -x, z; (iii) -x, -y, -z; (iv) -y, x-y, z; (v) x-y, x, -z; (vi) -x + 2/3,-y + 1/3,-z + 1/3]. Displacement ellipsoids are drawn at the 50% probability level.

**Fig. 2.**
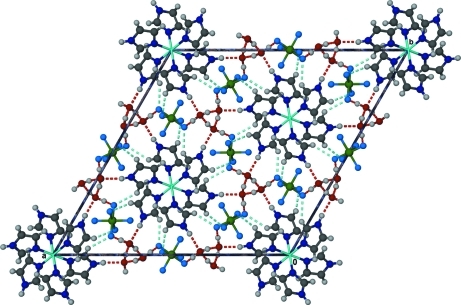
The crystal packing of (I) viewed down [001] with the extensive net of hydrogen bonds leading to a three-dimensional arrangement (the red dashed lines indicate strong hydrogen bonding and the blue weak ones).

## supplementary crystallographic information

### Comment

During the course of ongoing studies on Co^III^ complexes with heterocycles (Wojtczak *et al.*, 1990; Pazderski *et al.*, 2008) the title compound (I) was isolated. The crystal structure of (I) is built of mononuclear [Co(imidazole)_6_]^3+^ trications with the Co1 atom lying on Wyckoff position 3a (site symmetry 3), PF_6_^-^ anions with P atom lying on Wyckoff position 9 d (site symmetry 1) and lattice water molecules (Fig.1). It is worth mentioning that this is the first structure consisting of a hexakis(imidazole)-cobalt(III) cationic complex, whereas more than 15 structures consisting of a hexakis(imidazole)-cobalt(II) complex ion were reported. Furthermore, there are only two papers on the hexakis(imidazole)-cobalt(III) ion in solution, describing spectroscopic (Navon & Panigel, 1989) and kinetic properties (Wiśniewska & Kita, 2006) respectively. The Co^III^ ion has an almost ideal octahedral environment formed by six imidazole N atoms, with *cis*-N—Co—N angles of 88.68 (7)° and 91.32 (7)°. The planes of neighbouring imidazole rings are twisted from their usual perpendicular positions. This is the result of the extensive net of hydrogen bonds in the structure in which imidazole rings are involved (dihedral angle = 72.4 (6)°). The Co—N bond distance is equal to 1.961 (1) Å and as expected is slightly shorter than those reported for hexakis(imidazole)-cobalt(II) complexes with distances of 2.140±2.188 Å (Tong *et al.*, 2002). A similar relation of bond lengths was reported for Co^III^—N and Co(II)—N in hexaamminecobalt complexes (Kime & Ibers, 1969) with differences between those distances of 0.18 Å. The water molecules present in the crystal structure form helical chains (helix pitch = 7.016 (2) Å, *c* axis) similar to those observed in a trichlorophloroglucinol structure (Saha & Nangia, 2005). They are propagated in [001] directions by O1—H6···O1^i^ interactions (symmetry operation: -*y* + 2/3, *x*-*y* + 1/3, *z* + 1/3) with a distance of 2.913 (2) Å), which are stabilized by hydrogen bonding with the remaining molecular units (Table 1) giving in turn a three-dimensional assembly. The weak C—H···F interactions (Table 1) further stabilize the packing arrangement (Fig. 2).

### Experimental

A mixture of 11.84 g (174 mmol) of imidazole and 2.02 g (5 mmol) of Co(pyridine)_3_Cl_3_ was grinded for several minutes until the color changed from green to red. The mixture was subsequently dissolved in 100 ml of distilled water and the pH was adjusted to 2–3 by adding 3 *M* HCl. Then the mixture was diluted with water to a final volume of 2 l and passed through a Sephadex SP C-25 column starting with an aqueous 0.05 *M* HCl solution as eluent. The fraction obtained by using 0.2 *M* HCl was evaporated in a stream of cold air and the product [Co(imidazole)_6_]Cl_3_ was filtrated and washed with ethanol. 10 mg of this compound was placed in a 50 ml beaker and 20 ml of a saturated aqueous solution of NH_4_PF_6_ was added. Red crystals suitable for single-crystal X-ray analysis were obtained by slow evaporation.

### Refinement

Water H atoms were located in a difference map and refined with a restrained O—H distance of 0.96 (3) Å, whereas *U*_iso_(H) values were allowed to refine independently. The remaining H atoms were positioned geometrically, with C—H = 0.95 Å and N—H = 0.88 Å, and constrained to ride on their parent atoms with *U*_iso_(H) = 1.2*U*_eq_(C,*N*).

### Crystal data

**Table:**

[Co(C_3_H_4_N_2_)_6_](PF_6_)_3_·6H_2_O	*D*_x_ = 1.880 Mg m^−^^3^
*M**_r_* = 1010.43	Mo *K*α radiation, λ = 0.71073 Å
Trigonal, *R*3	Cell parameters from 3782 reflections
Hall symbol: -R 3	θ = 3.1–28.2°
*a* = 20.9911 (13) Å	µ = 0.77 mm^−^^1^
*c* = 7.0156 (9) Å	*T* = 100 K
*V* = 2677.1 (4) Å^3^	Block, red
*Z* = 3	0.25 × 0.25 × 0.21 mm
*F*(000) = 1530	

### Data collection

**Table:**

Bruker SMART APEX CCD area-detector diffractometer	1373 independent reflections
Radiation source: fine-focus sealed tube	1288 reflections with *I* > 2σ(*I*)
graphite	*R*_int_ = 0.026
ω scans	θ_max_ = 28.2°, θ_min_ = 3.1°
Absorption correction: multi-scan (*SADABS*; Sheldrick, 1997)	*h* = −22→27
*T*_min_ = 0.832, *T*_max_ = 0.856	*k* = −26→26
4893 measured reflections	*l* = −9→8

### Refinement

**Table:**

Refinement on *F*^2^	Primary atom site location: structure-invariant direct methods
Least-squares matrix: full	Secondary atom site location: difference Fourier map
*R*[*F*^2^ > 2σ(*F*^2^)] = 0.031	Hydrogen site location: inferred from neighbouring sites
*wR*(*F*^2^) = 0.083	H atoms treated by a mixture of independent and constrained refinement
*S* = 1.08	*w* = 1/[σ^2^(*F*_o_^2^) + (0.0444*P*)^2^ + 4.9022*P*] where *P* = (*F*_o_^2^ + 2*F*_c_^2^)/3
1373 reflections	(Δ/σ)_max_ < 0.001
98 parameters	Δρ_max_ = 0.54 e Å^−^^3^
3 restraints	Δρ_min_ = −0.31 e Å^−^^3^

### Special details

**Table:**

Geometry. All e.s.d.'s (except the e.s.d. in the dihedral angle between two l.s. planes) are estimated using the full covariance matrix. The cell e.s.d.'s are taken into account individually in the estimation of e.s.d.'s in distances, angles and torsion angles; correlations between e.s.d.'s in cell parameters are only used when they are defined by crystal symmetry. An approximate (isotropic) treatment of cell e.s.d.'s is used for estimating e.s.d.'s involving l.s. planes.
Refinement. Refinement of *F*^2^ against ALL reflections. The weighted *R*-factor *wR* and goodness of fit *S* are based on *F*^2^, conventional *R*-factors *R* are based on *F*, with *F* set to zero for negative *F*^2^. The threshold expression of *F*^2^ > σ(*F*^2^) is used only for calculating *R*-factors(gt) *etc*. and is not relevant to the choice of reflections for refinement. *R*-factors based on *F*^2^ are statistically about twice as large as those based on *F*, and *R*- factors based on ALL data will be even larger.

### Fractional atomic coordinates and isotropic or equivalent isotropic displacement parameters (Å^2^)

**Table:**

	*x*	*y*	*z*	*U*_iso_*/*U*_eq_	
Co1	0.0000	0.0000	0.0000	0.01001 (15)	
F2	0.25776 (5)	0.09189 (5)	0.20301 (16)	0.0242 (3)	
N1	0.05651 (7)	0.08788 (7)	0.15758 (19)	0.0126 (3)	
N3	0.14686 (7)	0.19255 (7)	0.2646 (2)	0.0161 (3)	
H3	0.1914	0.2302	0.2812	0.019*	
C5	0.02873 (8)	0.11667 (8)	0.2899 (2)	0.0155 (3)	
H5	−0.0213	0.0945	0.3285	0.019*	
C2	0.12841 (8)	0.13594 (8)	0.1462 (2)	0.0150 (3)	
H2	0.1618	0.1307	0.0653	0.018*	
C4	0.08453 (9)	0.18177 (8)	0.3556 (2)	0.0171 (3)	
H4	0.0810	0.2134	0.4464	0.020*	
P1	0.3333	0.1667	0.1667	0.01496 (16)	
F1	0.34563 (7)	0.13360 (7)	−0.02524 (19)	0.0421 (4)	
F3	0.37650 (6)	0.13458 (7)	0.2841 (2)	0.0402 (4)	
O1	0.27980 (6)	0.31811 (6)	0.37976 (18)	0.0203 (3)	
H6	0.3046 (15)	0.3048 (15)	0.473 (4)	0.052 (8)*	
H7	0.2625 (15)	0.3462 (14)	0.447 (4)	0.051 (8)*	

### Atomic displacement parameters (Å^2^)

**Table:**

	*U*^11^	*U*^22^	*U*^33^	*U*^12^	*U*^13^	*U*^23^
Co1	0.00877 (17)	0.00877 (17)	0.0125 (3)	0.00438 (9)	0.000	0.000
F2	0.0135 (4)	0.0191 (5)	0.0324 (6)	0.0026 (4)	0.0030 (4)	0.0038 (4)
N1	0.0126 (6)	0.0113 (6)	0.0144 (6)	0.0063 (5)	−0.0004 (5)	0.0004 (5)
N3	0.0148 (6)	0.0122 (6)	0.0179 (7)	0.0041 (5)	−0.0023 (5)	−0.0005 (5)
C5	0.0158 (7)	0.0158 (7)	0.0158 (8)	0.0085 (6)	−0.0001 (5)	−0.0015 (6)
C2	0.0137 (7)	0.0137 (7)	0.0171 (7)	0.0063 (6)	−0.0016 (5)	−0.0009 (5)
C4	0.0205 (8)	0.0153 (7)	0.0161 (8)	0.0095 (6)	−0.0020 (6)	−0.0023 (6)
P1	0.0104 (3)	0.0156 (3)	0.0172 (3)	0.0052 (2)	0.00177 (19)	0.0010 (2)
F1	0.0372 (7)	0.0354 (7)	0.0378 (7)	0.0062 (5)	0.0167 (5)	−0.0130 (5)
F3	0.0160 (5)	0.0422 (7)	0.0588 (9)	0.0118 (5)	0.0027 (5)	0.0283 (6)
O1	0.0192 (6)	0.0197 (6)	0.0207 (6)	0.0086 (5)	−0.0039 (5)	−0.0035 (5)

### Geometric parameters (Å, °)

**Table:**

Co1—N1	1.9605 (13)	C5—C4	1.361 (2)
Co1—N1^i^	1.9605 (13)	C5—H5	0.9500
Co1—N1^ii^	1.9605 (13)	C2—H2	0.9500
Co1—N1^iii^	1.9605 (13)	C4—H4	0.9500
Co1—N1^iv^	1.9605 (13)	P1—F1^vi^	1.5939 (12)
Co1—N1^v^	1.9605 (13)	P1—F1	1.5938 (12)
F2—P1	1.5985 (9)	P1—F2^vi^	1.5985 (9)
N1—C2	1.3340 (19)	P1—F3^vi^	1.6012 (11)
N1—C5	1.3854 (19)	P1—F3	1.6012 (11)
N3—C2	1.339 (2)	O1—H6	0.96 (3)
N3—C4	1.369 (2)	O1—H7	0.96 (3)
N3—H3	0.8800		
			
N1—Co1—N1^i^	88.68 (5)	N1—C5—H5	125.5
N1—Co1—N1^ii^	91.32 (5)	N1—C2—N3	110.48 (14)
N1—Co1—N1^v^	88.68 (5)	N1—C2—H2	124.8
N1—Co1—N1^iii^	180.00 (9)	N3—C2—H2	124.8
N1^v^—Co1—N1^iii^	91.32 (5)	C5—C4—N3	106.26 (14)
N1^v^—Co1—N1^i^	91.32 (5)	C5—C4—H4	126.9
N1^iii^—Co1—N1^i^	91.32 (5)	N3—C4—H4	126.9
N1^v^—Co1—N1^ii^	180.00 (9)	F1^vi^—P1—F1	180.0
N1^iii^—Co1—N1^ii^	88.68 (5)	F1^vi^—P1—F2	89.75 (6)
N1^i^—Co1—N1^ii^	88.68 (5)	F1—P1—F2	90.25 (6)
N1—Co1—N1^iv^	91.32 (5)	F1^vi^—P1—F2^vi^	90.25 (6)
N1^v^—Co1—N1^iv^	88.68 (5)	F1—P1—F2^vi^	89.75 (6)
N1^iii^—Co1—N1^iv^	88.68 (5)	F2—P1—F2^vi^	179.997 (1)
N1^i^—Co1—N1^iv^	180.00 (5)	F1^vi^—P1—F3^vi^	90.13 (8)
N1^ii^—Co1—N1^iv^	91.32 (5)	F1—P1—F3^vi^	89.86 (8)
C2—N1—C5	105.91 (12)	F2—P1—F3^vi^	90.15 (6)
C2—N1—Co1	126.96 (11)	F2^vi^—P1—F3^vi^	89.85 (6)
C5—N1—Co1	126.84 (10)	F1^vi^—P1—F3	89.86 (8)
C2—N3—C4	108.30 (13)	F1—P1—F3	90.14 (8)
C2—N3—H3	125.8	F2—P1—F3	89.85 (6)
C4—N3—H3	125.8	F2^vi^—P1—F3	90.15 (6)
C4—C5—N1	109.04 (14)	F3^vi^—P1—F3	180.0
C4—C5—H5	125.5	H6—O1—H7	105.7 (19)
			
N1^v^—Co1—N1—C2	−98.46 (15)	C2—N1—C5—C4	0.10 (17)
N1^i^—Co1—N1—C2	−7.11 (13)	Co1—N1—C5—C4	−174.02 (11)
N1^ii^—Co1—N1—C2	81.54 (15)	C5—N1—C2—N3	0.45 (17)
N1^iv^—Co1—N1—C2	172.89 (13)	Co1—N1—C2—N3	174.56 (10)
N1^v^—Co1—N1—C5	74.46 (10)	C4—N3—C2—N1	−0.82 (18)
N1^i^—Co1—N1—C5	165.81 (14)	N1—C5—C4—N3	−0.58 (18)
N1^ii^—Co1—N1—C5	−105.54 (10)	C2—N3—C4—C5	0.85 (18)
N1^iv^—Co1—N1—C5	−14.19 (14)		

Symmetry codes: (i) *y*, −*x*+*y*, −*z*; (ii) −*x*+*y*, −*x*, *z*; (iii) −*x*, −*y*, −*z*; (iv) −*y*, *x*−*y*, *z*; (v) *x*−*y*, *x*, −*z*; (vi) −*x*+2/3, −*y*+1/3, −*z*+1/3.

### Hydrogen-bond geometry (Å, °)

**Table:**

*D*—H···*A*	*D*—H	H···*A*	*D*···*A*	*D*—H···*A*
O1—H6···O1^vii^	0.96 (3)	1.98 (3)	2.913 (2)	163 (3)
N3—H3···O1	0.88	1.98	2.834 (2)	165
O1—H7···F3^viii^	0.96 (3)	2.10 (3)	2.945 (2)	146 (2)
C2—H2···F3^ix^	0.95	2.34	3.042 (2)	131
C4—H4···F1^x^	0.95	2.40	3.303 (2)	158

Symmetry codes: (vii) −*y*+2/3, *x*−*y*+1/3, *z*+1/3;  (viii) *x*−*y*, *x*, −*z*+1; (ix) −*x*+2/3, −*y*+1/3, −*z*+1/3; (x) −*x*+*y*+1/3, −*x*+2/3, *z*+2/3.
